# Reconstructing feedback in graduate medical education: development of the REFLECT scale to measure feedback delivery in medical residency training

**DOI:** 10.1186/s12909-023-04334-w

**Published:** 2023-05-17

**Authors:** Mehran Ilaghi, Sara Shafian, Adel Soltanizadeh, Ali Karamoozian, Maryam Okhovati, Sarah Aflatoonian

**Affiliations:** 1 Department of Medical Education, Education Development Center, Kerman University of Medical Sciences, Kerman, Iran; 2 Department of Biostatistics and Epidemiology, Kerman University of Medical Sciences, Kerman, Iran; 3 Medical Informatics Research Center, Institute for Future Studies in Health, Kerman University of Medical Sciences, Kerman, Iran

**Keywords:** Feedback, Medical education, Residency training, Assessment tool

## Abstract

**Background:**

Feedback plays a pivotal role in graduate medical education, where medical residents are expected to acquire a wide range of practical and professional competencies. Assessing the feedback delivery status is a preliminary step for educators to enhance the quality of feedback provided. This study aims to develop an instrument to assess the various aspects of feedback delivery in medical residency training.

**Methods:**

The fifteen-item REFLECT (Residency Education Feedback Level Evaluation in Clinical Training) questionnaire was developed. The content validity was evaluated according to a panel member consisting of fourteen clinical professors and medical education instructors. After evaluating the test-retest reliability, the questionnaire was distributed to a sample of 154 medical residents and was further assessed in terms of internal consistency and factor analysis.

**Results:**

Content validity analysis resulted in an appropriate content validity ratio and content validity index for the final 15 items. The test-retest reliability resulted in an ICC of 0.949 (95% C.I. 0.870–0.980), indicating excellent reliability. The Cronbach’s alpha for the 15-item questionnaire was α = 0.85, demonstrating good internal consistency. The factor analysis resulted in a four-factor structure: *“attitude towards feedback”*, *“quality of feedback”*, *“perceived importance of feedback”*, and *“reaction to feedback”*.

**Conclusions:**

REFLECT proved to a reliable tool that could be utilized as a quick assessment method of feedback delivery, making it a suitable aid for educational managers and faculties to design necessary interventions aiming to enhance the quantity and quality of feedback provided.

**Supplementary Information:**

The online version contains supplementary material available at 10.1186/s12909-023-04334-w.

## Introduction

Feedback is characterized as precise information delivered to a learner to encourage reflection on performance and focuses on both what was done and what the potential outcomes could be [1]. The development of a learners’ capacity to critically evaluate their own performance, and define future goals depends crucially on effective feedback [2]. In medical education, the importance of feedback cannot be overstated, as it is frequently referred to as the cornerstone of effective clinical teaching [3].

Feedback provision in healthcare education can be challenging, as feedback settings are diverse and encompass a broad spectrum of practical competencies and communication skills. In addition, it can be difficult for educators to provide feedback because they must take the learner’s psychosocial needs into account while making sure the feedback is accurate and honest [4]. Consequently, learners reportedly indicate that they receive less feedback, in terms of both quantity and quality, than what their educators believe they are giving, contributing to the so-called “*feedback gap*” [5, 6].

Assessing the feedback delivery status is a preliminary step for educators to address the gap and enhance the quality of feedback provided. Measuring the status of feedback provision in an educational environment where learners are overwhelmed with several theoretical and practical skills is essential to foster the learning process [7]. In medical education particularly, since the final product of training are physicians who are directly responsible for patient’s outcome, improving the feedback status will ultimately be a robust measure in enhancing the quality of patient care [8].

Due to the diversity of learning and working environments, several tools have so far been developed to assess different aspects of feedback based on the environment. For instance, the Feedback Environment Scale is a tool developed by Steelman et al. that has been originally used in contexts other than medical education, namely in organizations to support managers in terms of training and coaching [9]. Additionally, Ashford has proposed a measure of feedback-seeking in organizations [10]. Other attempts have aimed to develop instruments to measure the learning environment. As an example, the Surgical Theatre Educational Environmental Measure (STEEM) has been developed to assess the quality of the learning environment in operating theatre perceived by surgical trainees [11]. Similarly, the Anesthetic Theatre Educational Environment Measure (ATEEM) has been designed to measure the educational environment for trainee anesthetists [12] and later on, the Dundee Ready Educational Environment Measure (DREEM) has been proposed as a generic tool for measuring students’ perceptions of undergraduate health professions curricula [13]. According to the existing literature on the instruments assessing feedback, most scales have been developed to evaluate the educational environment, and there are few validated and reliable scales to exclusively assess different aspects of feedback delivery in residency training.

Considering the significance of high-quality feedback provision in graduate medical education, in the present study, we aimed to develop a valid and reliable scale to assess feedback delivery in residency training.

## Methods

### Participants

The current study was performed on medical residents who had been studying in Kerman University of Medical Sciences in the 2021–2022 academic year, and had spent at least 6 months since their admission. According to the Morgan table, considering the total number of registered residents at the time of study, the minimum required sample size was determined to be 154 [14]. According to the number of residents in each specialty, a quota sampling was used to ensure that a minimum of participants in each specialty were enrolled in the study. Residents were allowed to participate during working hours, confidentiality was guaranteed and disclosure of results was promised. All participants were enrolled upon receiving informed consent. Overall, a total of 154 medical residents (mean ± SD age: 31.06 ± 3.35; 61.7% female) from 17 residency program specializations were enrolled in the study. The majority of participants were under training in internal medicine (n = 31), gynecology and obstetrics (n = 14), radiology (n = 13), psychiatry (n = 13), general surgery (n = 12), cardiology (n = 12), and neurology (n = 11) residency programs. Other specializations included pediatrics (n = 9), orthopedics (n = 6), neurosurgery (n = 6), urology (n = 5), pathology (n = 5), dermatology (n = 4), radiation oncology (n = 4), anesthesiology (n = 3), otorhinolaryngology (n = 3), and ophthalmology (n = 3).

### Questionnaire design

In order to generate the most relevant items assessing the quality of feedback delivery in medical residency training, a comprehensive review of the literature was performed. Databases, including Pubmed/Medline, WoS, ERIC, Scopus, and Google Scholar, as well as domestic databases, such as SID and Magiran, were searched using appropriate keywords. The initial search strategy was not limited to the field of medical education; rather, the search encompassed general scales developed to assess various aspects of feedback in non-clinical settings. In the next step, the search was narrowed down to the studies conducted on feedback delivery in medical education. Therefore, studies addressing the feedback provision in medical education, as well as previously developed tools for evaluating feedback in other settings were taken into account. Relevant studies were evaluated by four reviewers (M.I, A.S, S.A, and S.S).

A preliminary set of items addressing various aspects of feedback in postgraduate medical education were independently proposed by the reviewers. The authors set a meeting and agreed upon the items by consensus. Subsequently, an expert panel consisting of medical education instructors were asked to provide further suggestions, either to modify or replace items. Eventually, a total of eighteen items were included in the first draft of the questionnaire, which were subsequently tested for psychometric properties. The questionnaire was named REFLECT (Residency Education Feedback Level Evaluation in Clinical Training). Each item was scored based on a 5-point Likert scale (completely disagree = 0, completely agree = 4). Accordingly, as the proposed items highlighted a positive aspect of feedback, a higher score indicates a more positive attitude towards the essence of feedback or the quality of feedback received. Considering the fact that one item (no. 14) addressed the emotional response towards negative feedback, where a higher score highlighted a dysfunctional response against the received feedback, a reverse scoring was considered for this item.

### Data analysis

All the analyses were performed using the Statistical Package for the Social Sciences (SPSS) software (version 22.0. SPSS, Inc., Chicago, IL, USA) and Mplus 7.0 for Windows. Assessment of content validity, reliability, and factor analysis were done as follows:

#### Content validity

To assess the content validity of the developed instrument, the questionnaire was distributed to a panel consisted of seven clinical professors and seven medical education instructors. The content validity ratio (CVR) and content validity index (CVI) were measured as previously proposed by Lawshe [15] and Waltz & Bausell [16]. In brief, the CVR was calculated using the following formula where n_e_ is the number of panel members who indicate an item is essential, and N is the total number of panel members:

$$CVR = \frac{{ne - N/2}}{{N/2}}$$

According to the Lawshe table, given that there were fourteen members in our panel, a CVR lower than 0.51 resulted in the elimination of the item. The item-level CVI (I-CVI) was measured based on the proportion of experts giving the item a relevance rating of 3 or 4, on a scale of 1 to 4. The item was retained if it got an I-CVI more than 0.79.

#### Reliability

To assess the test-retest reliability, the questionnaire was distributed twice in a predetermined subsample of participants (20 residents) with a two-week time interval between the test, and the intraclass correlation coefficient (ICC) was measured. As the same questions were being asked, it was believed that this time period would be sufficient to prevent any interference with the results due to the clinical rotation changes of residents. The inter-item correlation and Cronbach’s alpha were measured to test the internal consistency of the questionnaire.

#### Factor analysis

Exploratory factor analysis (EFA) was performed to specify the structure and underlying dimensions of the scale. The Kaiser-Meyer-Olkin (KMO) measure of sampling adequacy and Bartlett’s sphericity test were done to assess the eligibility to EFA. The EFA was conducted through the principal component method to estimate the factor loadings and specificity, and by adopting the varimax rotation method in a correlation matrix. The scree plot was used to determine the number of factors to retain in an EFA [17].

### Ethical considerations

This study has been conducted under the approval of the Ethics Committee of Kerman University of Medical Sciences (Ethics code: IR.KMU.REC.1400.646).

## Results

### Content validity

The CVR and CVI of the preliminary items were calculated according to a panel consisted of fourteen members. Based on the calculated indices, out of the initial eighteen items, three were discarded due to a low CVR or CVI, and the other fifteen items were retained. Briefly stated, the eliminated items were as follows: “*feedbacks modify the way I think”*, “*The feedbacks I receive, specifically concern one or a limited number of subjects and are not general”*, and “*I generally receive positive feedback from my professors or colleagues*”. The CVR and CVI of the retained items are demonstrated in Table 1.

**Table 1:** Content validity indices of the items

No.	Item	CVR	CVI
1	Feedbacks improve my clinical performance.	1.00	1.00
2	Feedbacks improve my professional behavior.	0.86	0.93
3	Feedbacks increase my academic motivation.	0.71	0.93
4	Feedbacks are influential in making me a better specialist in the future.	0.57	0.86
5	I consider my fellow or senior residents to be a reliable source of delivering feedback to me.	0.71	0.79
6	Feedbacks are provided to me at the appropriate time.	0.71	0.93
7	Feedbacks are provided to me at the appropriate place.	0.71	0.86
8	The provided feedback is completely clear.	0.86	0.93
9	When receiving feedback, a solution is provided to improve my performance.	0.86	1.00
10	The faculty spend sufficient time getting to know me, evaluating me, and providing feedback.	0.57	0.93
11	In my opinion, the faculty have sufficient skills and follow an appropriate framework in providing feedback.	0.57	0.86
12	I consider the feedback from faculty to be necessary and important for my progress.	0.71	0.93
13	In case I do not find the received feedback sufficient, I personally seek feedback from professors or other residents.	0.71	0.79
14	Receiving negative feedback makes me feel stressed, embarrassed, or humiliated.	0.57	0.79
15	Receiving positive feedback makes me feel good.	0.57	0.79

### Reliability

The test-retest reliability resulted in an ICC of 0.949 (95% C.I. 0.870–0.980), indicating excellent reliability. The Cronbach’s alpha for the 15-item questionnaire was α = 0.85, demonstrating good internal consistency.

### Factor analysis

The KMO index returned a value of 0.845, indicating that the data were suitable for factor analysis. Bartlett’s sphericity test was significant (χ²=887.85, df-105, p < 0.001) allowing the EFA to be performed.

**Fig. 1:**
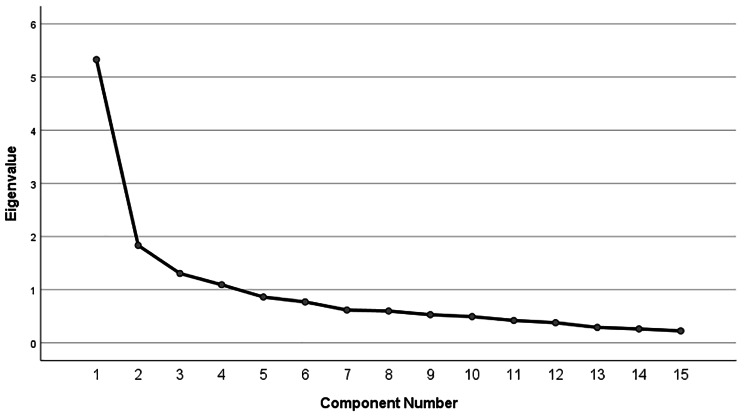
Scree plot of the extracted factors

Table 2 demonstrates the explained variance according to varimax rotation and the number of factors according to eigenvalues in the EFA. According to the results, the use of four factors explained 63.72% of the total variance (Table 2). The Scree plot supported a four-factor solution as well (Fig. 1).

**Table 2:** Total variance explained according to varimax rotation

Component	Initial eigenvalues			Rotation sums of squared loadings		
	Cumulative %	% of Variance	Total	Cumulative %	% of Variance	Total
**1**	35.524	35.524	5.329	24.474	24.474	3.671
**2**	47.743	12.219	1.833	46.425	21.951	3.293
**3**	56.445	8.702	1.305	55.427	9.003	1.350
**4**	63.722	7.277	1.092	63.722	8.295	1.244
**5**	69.466	5.744	0.862	-	-	-
**6**	74.592	5.126	0.769	-	-	-
**7**	78.705	4.114	0.617	-	-	-
**8**	82.693	3.987	0.598	-	-	-
**9**	86.221	3.528	0.529	-	-	-
**10**	89.506	3.285	0.493	-	-	-
**11**	92.305	2.800	0.420	-	-	-
**12**	94.827	2.522	0.378	-	-	-
**13**	96.762	1.934	0.290	-	-	-
**14**	98.503	1.741	0.261	-	-	-
**15**	100.000	1.497	0.225	-	-	-

Table 3 demonstrates the allocation of items to appropriate factors after varimax rotation according to the EFA. Based on the results, items “1” to “5” were categorized to the *“attitude towards feedback”* factor, items “6” to “11” fell into to the *“quality of feedback”* factor, items “12” and “13” were categorized to the *“perceived importance of feedback”* factor, and items “14” and “15” were related to the *“reaction to feedback”* factor (Table 3).

**Table 3:** Allocation of items to appropriate factors according to EFA

No.	Item	Factors			
		Attitude Towards Feedback	Quality of Feedback	Perceived importance of Feedback	Reaction to Feedback
**1**	Feedbacks improve my clinical performance.	**0.782**			
**2**	Feedbacks improve my professional behavior.	**0.785**			
**3**	Feedbacks increase my academic motivation.	**0.799**			
**4**	Feedbacks are influential in making me a better specialist in the future.	**0.770**			
**5**	I consider my fellow or senior residents to be a reliable source of delivering feedback to me.	**0.603**			
**6**	Feedbacks are provided to me at the appropriate time.		**0.719**		
**7**	Feedbacks are provided to me at the appropriate place.		**0.608**		
**8**	The provided feedback is completely clear.		**0.534**		
**9**	When receiving feedback, a solution is provided to improve my performance.		**0.669**		
**10**	The faculty spend sufficient time getting to know me, evaluating me, and providing feedback.		**0.839**		
**11**	In my opinion, the faculty have sufficient skills and follow an appropriate framework in providing feedback.		**0.770**		
**12**	I consider the feedback from faculty to be necessary and important for my progress.			**0.840**	
**13**	In case I do not find the received feedback sufficient, I personally seek feedback from professors or other residents.			**0.542**	
**14**	Receiving negative feedback makes me feel stressed, embarrassed, or humiliated.				**0.806**
**15**	Receiving positive feedback makes me feel good.				**− 0.695**

Factor intercorrelation matrix is presented in Table 4, where the *“attitude towards feedback”* factor significantly correlated with all other factors, and a significant correlation between the “quality of feedback” and *“perceived importance of feedback”* was present (Table 4).

**Table 4:** Factor intercorrelation matrix from EFA

Factors	Attitude Towards Feedback	Quality of Feedback	Perceived importance of Feedback	Reaction to Feedback
**Attitude Towards Feedback**	-			
**Quality of Feedback**	0.48 **	-		
**Perceived importance of Feedback**	0.28 **	0.29 **	-	
**Reaction to Feedback**	0.21 *	0.06	0.14	-

**p-value < 0.01, *p-value < 0.05

## Discussion

While assessing the perspective of medical residents towards an educational issue, utilizing valid instruments that are brief enough to consider their time and work overload while guaranteeing their participation could be of significant help for instructors to find out the challenges of clinical training and to address further the gap between the current status and desired level. Due to the scarcity of a valid and reliable tool to evaluate the feedback delivery status, in the present study we explored the validity and reliability of a scale focusing on feedback evaluation in residency program: a brief 15-item scale called REFLECT that could be utilized to assess various dimensions of feedback delivery in graduate clinical training.

REFLECT proved to be a short, easy-to-use instrument that could provide insight to clinical instructors about what the attitude of medical residents towards feedback is, how they evaluate the quality of feedback provided to them, how much getting feedback or feedback seeking is important to them, and finally what their reaction to feedback is. In addition to its validity, REFLECT demonstrated excellent reliability (ICC = 0.949) and good internal consistency (Cronbach’s alpha = 0.85), which is comparable to previously designed instruments designed to measure learning environments or address feedback in other settings [11, 12, 18]. To make the best out of the developed instrument, we aimed to provide items that assess different aspects of feedback delivery pertinent to graduate clinical training. Accordingly, the factor analysis yielded a four-factor solution to the questionnaire. Each factor pertains to a specific dimension of feedback delivery which has been of utmost importance according to previous literature. Herein, based on the existing literature, we discuss how each factor obtained through REFLECT might be utilized to address current debates on feedback delivery topic and provide faculties with extra insight into the current status of feedback delivery in their institutions.

*“Attitude towards feedback”* evaluates how a medical resident finds the role of feedback in forming his professional identity. This encompasses the impact of feedback on clinical performance, professional behavior, academic motivation, and generally making a better specialist after the training ends. A positive attitude towards feedback motivates the learner to maximize the value of the feedback given. A bulk of evidence suggests that high-quality feedback positively impacts the knowledge, attitude, and skills of learners in clinical setting. For instance, in a recent systematic review and meta-analysis on the effectiveness of the use of different feedback modalities, de Almeida et al. demonstrated that feedback has a positive influence on the education-learning process of students in clinical setting [19]. Moreover, the *“Attitude towards feedback”* factor addresses the attitude of medical residents toward the reliable source of getting feedback. It is traditionally believed that in clinical training, providing feedback is exclusive to a clinical professor. However, in graduate medical education, residents spend most of their clinical encounters supervised by their peers or senior residents. A positive attitude towards the feedback obtained from fellow residents substantially impacts the flow of feedback delivery within clinical shifts while encouraging the resident to devote time and effort to providing feedback to his peers as well. Previous studies have also underscored the integration of peer feedback into both medical student and resident education. For instance, Sheahan et al. demonstrated that structured peer feedback was comparable to faculty feedback in the acquisition of surgical skills [20]. In another survey of internal medicine residents, 72% of the residents felt that peers could provide valuable feedback. Interestingly, more than 80% believed that peers observe behaviors that are not seen by attending faculty [21]. Building on this evidence, it is generally believed that peer feedback is a potentially useful tool to promote clinical excellence in medical education [22].

To ensure that residents are receiving high-quality feedback, it is crucial to assess whether feedback is delivered in an appropriate form. In this regard, in the *“quality of feedback”* factor, we aimed to assess the most important characteristics of proper feedback. It should be noted that the perception of the learner regarding the quality of feedback might be completely different from his instructors. For example, in a study by Liberman and colleagues, it was reported that surgery residents and their attending surgeons had significantly different perceptions of feedback. Interestingly, almost 90% of surgeons felt that they were successful at giving effective feedback, while nearly 17% of the residents agreed [1]. This gap could be bridged if the perception of residents from the quality of received feedback is clearly defined. On this basis, the faculty could be informed about the shortages and pitfalls of the feedback they are providing.

Although there is not a definite consensus on how effective feedback should be provided, plenty of tips and guidelines are suggested for providing high-quality feedback in a clinical environment [23, 24]. The time of feedback delivery is an important feature defining the effectiveness of feedback. Feedback should be provided at the right time and on a regular basis. Most instructors argue that, to ensure feedback has its maximum efficacy, it should be provided right after the performance is observed [1, 25]. Additionally, providing feedback in the right place is another factor contributing to an effective feedback. For instance, a physical space where private feedback exchange sessions are held could foster the process [26]. Moreover, feedback should be clear and precise so that the learner completely understands what his instructor refers to and in which part of his performance he needs support. More importantly, the feedback provider should come up with a practical solution, referred to as an action plan, to better guide the learner on how to compensate for his shortcomings [23, 27]. Last but not least, the amount of time a clinical professor devotes to maintaining a relationship with the learner, evaluating his performance, and providing feedback is another determinant of high-quality feedback. It has been suggested that the quality of feedback centers on the degree of contact between the clinical professor and resident [28]. Residents often express that the lack of time devoted to observation by a clinical instructor diminishes the credibility of the feedback he provides. They believe that increased interaction with their professors makes them feel more at ease and eager to seek feedback. On the other hand, faculty concur that spending more time with residents enables them to provide more individualized feedback [28].

The *“perceived importance of feedback”* further evaluates the degree to which the learner finds feedback necessary for his progress and the extent he exhibits feedback-seeking behavior. The focus of postgraduate medical education has recently evolved to a learner-focused approach with a greater emphasis on the learner’s role in the feedback exchange [29, 30]. Thereby, an effective feedback exchange necessitates that the learners be active seekers of feedback. The potential gap between the perceived importance of feedback and feedback seeking gives a clue that there are barriers that prevent the resident from seeking feedback which should be further investigated by the clinical educators.

Finally, *“reaction to feedback”* corresponds to how a resident reacts to the feedback provided. Basically, the fear of receiving negative feedback is considered a significant barrier to feedback exchange [31, 32]. Learners often feel embarrassed when they receive negative feedback, while their performance is reinforced when positive feedback is provided to them. On the faculty side also, studies have repeatedly indicated that professors might avoid giving negative feedback because they fear that they hurt the learner’s feelings or because they tend to keep a decent relationship with their students [33, 34]. Therefore, to make the best out of feedback, the emotional response of the learner should be taken into account. In line with this, several feedback delivery techniques (e.g., sandwich feedback) have been proposed to balance the burden of negative feedback on the learner [35]. In this regard, the learner considers negative feedback as constructive criticism for his personal development rather than merely feeling fear or humiliation.

The current study benefits from several strengths. First, a reasonable number of residents from all residency specialties participated. Moreover, the brevity of the developed scale makes it possible to consider the time and work overload of residents, thus enabling it to be used in large scales. Despite the efforts and rigor of the primary research, we faced some minor limitations in this study. We collected data from four public educational academic hospitals of the Kerman university of medical sciences in one geographic region of Iran. Therefore, the findings may not be generalized to other medical schools in other countries. Future studies are needed to validate translated versions in different contexts.

## Conclusions

Overall, this study presented a brief scale to assess various dimensions of feedback delivery to medical residents in a clinical setting. REFLECT could be utilized as a quick assessment method of feedback delivery, making it a suitable aid for educational managers and clinical professors to intervene appropriately in order to enhance the quantity and quality of feedback provided. There is a need for future research on the use of this scale, and its applicability to different residency programs.

## Electronic supplementary material

Below is the link to the electronic supplementary material.

Supplementary Material 1

## Data Availability

The datasets used or analyzed during the current study are available from the corresponding author upon reasonable request.

## List of abbreviations

REFLECT: Residency Education Feedback Level Evaluation in Clinical Training
CVR: Content validity ratio
CVI: Content validity index
ICC: Intraclass correlation coefficient
EFA: Exploratory factor analysis
