# Supplementary material for: Reconstructing feedback in graduate medical education: development of the REFLECT scale to measure feedback delivery in medical residency training
Source: BMC Med Educ. 2023 May 17;23:344. doi: 10.1186/s12909-023-04334-w (PMC10193812; doi:10.1186/s12909-023-04334-w)
Supplement: Supplementary file 1 — Supplementary Material 1 [file 12909_2023_4334_MOESM1_ESM.docx]

**The REFLECT Questionnaire**

| **Item** | | **Completely agree** | **Agree** | **No idea** | **Disagree** | **Completely disagree** |
| --- | --- | --- | --- | --- | --- | --- |
| **1** | Feedbacks improve my clinical performance. |  |  |  |  |  |
| **2** | Feedbacks improve my professional behavior. |  |  |  |  |  |
| **3** | Feedbacks increase my academic motivation. |  |  |  |  |  |
| **4** | Feedbacks are influential in making me a better specialist in the future. |  |  |  |  |  |
| **5** | I consider my fellow or senior residents to be a reliable source for delivering feedback to me |  |  |  |  |  |
| **6** | Feedback are provided to me at the appropriate time. |  |  |  |  |  |
| **7** | Feedback are provided to me at the appropriate place. |  |  |  |  |  |
| **8** | The provided feedback is completely clear. |  |  |  |  |  |
| **9** | When receiving feedback, a solution is provided to improve and improve my performance. |  |  |  |  |  |
| **10** | The faculty spend a sufficient amount of time to get to know me, evaluate me and provide feedback. |  |  |  |  |  |
| **11** | In my opinion, the faculty have sufficient skills in providing feedback and follow an appropriate framework. |  |  |  |  |  |
| **12** | I consider the feedback from faculty to be necessary and important for my progress. |  |  |  |  |  |
| **13** | In case I do not find the received feedback sufficient, I personally seek feedback from professors or other residents. |  |  |  |  |  |
| **14** | Receiving negative feedback makes me feel stressed, embarrassed or humiliated. |  |  |  |  |  |
| **15** | Receiving positive feedback makes me feel good. |  |  |  |  |  |
